# Rambles in Military Hospitals

**Published:** 1863-01

**Authors:** R. M. Lackey

**Affiliations:** Louisville, Ky.


### RAMBLES IN MILITARY HOSPITALS.

By R. M. LACKEY, M. D.

Some weeks since I visited the military hospital at Camp House, near the city of Pittsburgh, Pa. The building was constructed expressly for hospital purposes, and was a low structure nearly square, with only one large ward, and that poorly ventilated.

I am almost ashamed of my brethren of the profession and my fellow citizens, when I state the condition in which I found that hospital, and the patients in it. In the same ward were wounded men and those sick with fevers and contagious diseases, and in the whole room, intended to accommodate eighty or a hundred patients, there were not more than ten or twelve cots, bedsteads or bunks of any kind. The men were lying on the filthy floor, some with a straw mattrass under them and a part of them with nothing under them but a blanket, nearly all had their dirty clothes on that they came in with; everything was filthy and disordered. I did not make the acquaintance of the Surgeon who had this institution in charge, and I am glad I did not, for I might have been tempted to mention his name to his disadvantage and with no advantage to me or any one else.

There was in this hospital some cases of "sore throat," and one of the assistants informed me, that Chlorate of Potash was *the* remedy.

The military hospitals in Cincinnati, Ohio, are in excellent condition. There are not very extensive hospital accommodations immediately in the city, but at Camp Dennison, fifteen miles out, I am informed there are twelve or fifteen hundred sick and wounded soldiers in hospital.

Louisville, Ky., and the towns in the vicinity of the Falls, boast of more than thirty hospitals. Some of the buildings used are very well adapted for hospitals, several of the public and school buildings having been appropriated, and the necessary additions made to them. Each of these hospitals is in charge of a Surgeon, who has two or three assistants, most of these surgeons and assistants are private physicians and surgeons, residents of the city, who are employed on contract.

There is but a small proportion of very sick men here. The army is now so far distant, that the sickest men cannot bear transportation to this place, and those who are very seriously wounded cannot be brought here for the same reason, so that it is not until a severe wound is nearly well that you can see it in the hospitals here.

A large number of the cases found in the hospitals here are chronic, and the patients are subjects for discharge from the service. Chronic Diarrhœa, as is usual, predominates over any one disease in the number of its victims, and the surgeons here, as elsewhere, are not decided upon any one course of treatment as the best in all cases. Some say purging and vomiting cure more than any other means they have employed, others say give blue mass and morphine, and others again use Fowler's Solution with success in many cases. Ether and laudanum are given, as many assert, with benefit. One surgeon in New Albany, a very intelligent man, says that his diarrhœa patients all get well in the kitchen. If the patient is able to spend a part of his time in the cooking apartment, this surgeon regards his recovery as almost certain. This remedy should be employed by surgeons, as I am persuaded, that in many cases, it may be the means of restoring patients who have become prostrated by disease, and kept in that weakened condition by constant confinement in the wards of a hospital.

My attention was called to a case of tetanus that occurred in one of the hospitals here, a short time since. It followed a gunshot wound of the foot. The man was put upon large doses of Indian Hemp, (1 gr. three times a day of the solid ext.), and was kept upon this treatment throughout—the case terminating favorably. The Surgeon, who called my attention to this case, stated that out of five or six similar cases that had occurred in that hospital since it opened, more than a year ago, this was the only one that had terminated favorably, and the only one that had been treated with the hemp.

In Hospital No. 5, I saw two very interesting cases of gunshot wounds. The men were both wounded at Shiloh, on the 7th of April last. I made the following notes on these cases:

Jno. Winter, of 39th Regt. Ind. Vols., was wounded by a Minie ball in the middle third of thigh. The ball passed from within outward, fracturing the femur, and lodged beneath the skin and superficial fascia on the outside of the thigh, it was removed soon after the wound was received by cutting through the skin and fascia. The man showed me the ball, which was such as are shot from the Enfield rifle; the ball was flattened on one side, otherwise it was of its original size and shape.

In some way, this limb escaped amputation, and for some reason the man did not die.

Present condition—His general health tolerably good, has no diarrhœa, appetite is good, and when I saw him he was in good spirits. The limb is much swollen, and has been in that condition ever since he came to this hospital, which was about the 15th of April last.

The wound is discharging considerable pus and some pieces of bone. There is some firmness at the point of fracture; the limb can be moved by the nurse without causing pain, and the patient himself can turn the limb to one side or the other when he wishes to lie on his side. This man is now in much better condition, in every respect, than ever before since the wound was received, and he may get well with a leg somewhat inferior to a wooden one.

Chas. Cridler, of the 15th Regt. U. S. Regulars, was wounded by a Minie ball in the knee-joint, on the 7th of April, at the battle of Shiloh. The ball passed from without inwards, and lodged beneath the skin, on the inner side of the patella.

The ball was removed, but for some unaccountable reason, the limb was not amputated. The man's general condition is not good; he has a poor appetite, bowels are frequently deranged, he has abscesses about the parotid region, that cause great suffering. The limb is painful also, and much swollen. The wound discharges a large amount of unhealthy matter and small pieces of bone. This man refuses to have his limb amputated upon any condition whatever, and in his present reduced condition it would hardly be justifiable. The man may live two or three months yet, and it is contrary to the usual course of such cases that he has lived until this time.

These are the only cases of wounds of this nature I have met with in which amputation was not performed, and the patients have survived the injury so long as these men have. The indications are that the man with the fracture of the thigh will recover with a limb that will be of some use to him. Still I do not regard this case as an argument in favor of conservative surgery, in this class of wounds.

The hospital surgeons in this city, and many other places, have formed "Associations," and meet once a week for mutual improvement. Notes are kept of all cases of unusual interest, and the cases reported at a meeting of the Association. Attention is paid also to the study of pathological anatomy. These meetings of intelligent members of the profession with such ample means of investigation, promise to contribute much to the advancement of military medicine and surgery.

LOUISVILLE, Ky., Dec. 2, 1862.

